# One Health approach to *Coxiella burnetii*: first serosurvey of owners and dogs living on oceanic islands and mainland seashore areas of Brazil

**DOI:** 10.3389/fpubh.2025.1643457

**Published:** 2025-08-13

**Authors:** Aaronson Ramathan Freitas, Danilo Alves de França, Louise Bach Kmetiuk, Rafaella Martini, Ruana R. Delai, Claudia Turra Pimpão, João Henrique Perotta, Ivan Roque de Barros-Filho, Fabiano Borges Figueiredo, Rogério Giuffrida, Vamilton Alvares Santarém, Helio Langoni, Alexander Welker Biondo

**Affiliations:** ^1^Graduate College in Veterinary Sciences, Federal University of Paraná (UFPR), Curitiba, Brazil; ^2^Department of Animal Production and Preventive Veterinary Medicine, São Paulo State University (UNESP), Botucatu, Brazil; ^3^Center of Environmental Health, City Secretary of Health, Curitiba, Brazil; ^4^Department of Physiology, Federal University of Paraná (UFPR), Curitiba, Brazil; ^5^Department of Animal Science, Pontifical Catholic University of Paraná (PUCPR), Curitiba, Brazil; ^6^Carlos Chagas Institute (ICC), Oswaldo Cruz Foundation (FIOCRUZ), Curitiba, Brazil; ^7^Graduate College in Animal Sciences, University of Western São Paulo (UNOESTE), Presidente Prudente, Brazil

**Keywords:** infectious diseases, public health, Q fever, serological analysis, zoonosis

## Abstract

*Coxiella burnetii*, the causative agent of Q fever, a zoonotic pathogen with primarily airborne transmission and diverse host species, have been reported in serosurveys of vulnerable populations worldwide. Although vulnerable populations from oceanic islands and mainland seashore areas in Brazil may be also exposed *C. burnetti*, no study to date has concomitantly assessed owners and dogs in such environmental setting. Accordingly, the present study aimed to assess the *C. burnetii* seroprevalence in owners and their dogs from oceanic islands and mainland seashore areas of southern Brazil. Overall, 5/335 (1.49%) humans and none/352 (0%) dogs of three oceanic islands and two mainland coastal areas were seropositive to anti-*Coxiella burnetii* antibodies by Indirect Immunofluorescence Assays (IFA). Such low seroprevalence may have been consequence of the livestock absence of these environmentally preserved islands, in addition to a major geographical barrier to airborne dispersal in southern Brazilian seashore mainland areas due to the rainforest (Serra do Mar) mountains. Finally, absence of dog seropositivity herein, suggesting that seropositive humans were exposed to *C. burnetti* outside these islands and seashore mainland areas, may provide new insights of *C. burnetii* transmission, disease cycle and prevention.

## 1 Introduction

*Coxiella burnetii*, a generalist zoonotic bacterium and the causative agent of Q fever, has presented a challenge for researchers worldwide, with disease epidemiology remaining to be fully established (1). *C. burnetii* has been mostly detected and associated with ruminant livestock, pets and wildlife, indicating the importance of animal surveillance and monitoring for diagnosis, control and prevention to human infection (2). Despite the *C. burnetii* transmission may occur by different routes, airborne transmission from bacterial source to susceptible hosts has been considered the main pathway (2). Large amounts of *Coxiella* may be released into the environment during the livestock abortion of infected goats, sheep and cattle, with bacterial spores traveling up to 30 km when favored by windy environment (1, 2).

*C. burnetii* seropositivity have been reported in vulnerable and overexposed populations in Brazil, and included park employees, zookeepers, and animal service workers (3), women inmates and correctional officers (4), indigenous individuals and their dogs (5), police officers and working dogs (6), *quilombola* individuals and their dogs (7). In overall, human serosurveys in Brazil have shown 129/604 (21.4%) seropositive individuals living in urban centers near to livestock (8), 44/200 (22.0%) rural *quilombola* (former slavery) individuals (7), 1/18 (5.5%) police officers and 9/30 (30%) working dogs (9), 25/309 (8.1%) park rangers, zookeepers, and animal service workers, and 139/413 (33.7%) female inmates in 2020 and 68/166 (41.0%) in 2021 (9). In addition, the human:dog seropositivity ratio in Brazil has varied from 1:3.6 (8/893, 0.90%; 1/406, 0.25%) in indigenous communities (5), 1:5.5 (1/18, 5.5%; 9/30, 30.0%) in K9 police units (6), to 44:1 (44/200, 22.0%; 1/20, 0.5%) in rural *quilombola* communities (7), suggesting different concomitant human:dog exposure, multiple transmission pathways and potential role of wildlife reservoirs. The only other concomitant human:animal serosurvey outside Brazil was performed in Afghanistan, showing 63.9% seroprevalence in humans, 43.4% in sheep, 52.7% in goats and 5.2% in cattle, with no dog surveillance at the time (10).

Although vulnerable populations from oceanic islands and mainland seashore areas in Brazil may be also exposed *C. burnetti*, no study to date has concomitantly assessed humans and dogs in such environmental setting. Accordingly, this study aimed to assess the seroprevalence *C. burnetii* in owners and their dogs living in oceanic islands and mainland seashore areas of southern Brazil.

## 2 Methods

### 2.1 Study design

The present study was a cross-sectional seroepidemiological survey directed to owners and their dogs living in oceanic islands and mainland coastal areas in the Paraná state, southern Brazil, between July 2019 and February 2020.

### 2.2 Ethical statement

The study herein was approved by the Animal Use Ethics Committee of the Federal University of Paraná (protocol 036/2021) and the Human Health Ethics Committee of the Ministry of Health (protocol 84756324.0.0000.0020).

### 2.3 Study area

The serological survey was conducted in three oceanic islands (Superagui Island, Mel Island, and Peças Island) and two mainland coastal areas (Guaraqueçaba and Pontal do Paraná cities), all located in the Paraná state, southern Brazil. The three islands were part of two major unit conservation areas named Ilha do Mel State Park and Superagui National Park, which are part of the largest continuous preserved area of the Atlantic Rainforest biome in Brazil (11).

The largest of the three islands, Mel Island (25°2′32.4″ S and 48° 8′ 15.1″ W) harbors two important environmental conservation areas, the Ecological Station and the State Park, both protected by environmental laws and covering 93.4% of its 2,762 island hectares (11). Despite its small permanent population, with around 1,100 inhabitants, the island ranked at the time as the second most visited state tourist destination, receiving around 200,000 visitors annually (12, 13). The local residents were distributed in five villages, mainly relying on tourism for their livelihood (14). The Superagui Island (25°7′27.1″S and 48°4′43.6″W), part of the Superagui National Park, has been a conservation unit located nearby artisanal fishing communities, with around 700 inhabitants and 350 dogs living in the main island community at the time (14). The smallest of the three islands (25°7′36.5″S and 48°0′06.5″W), Peças Island was a full environmentally protected area at the Superagui National Park, mostly due to its importance as a dolphin breeding and nursery area (14). Colonized during the slavery trade period, this island was home to ~350 residents, who relied on artisanal fishing and tourism for living.

**Figure 1:**
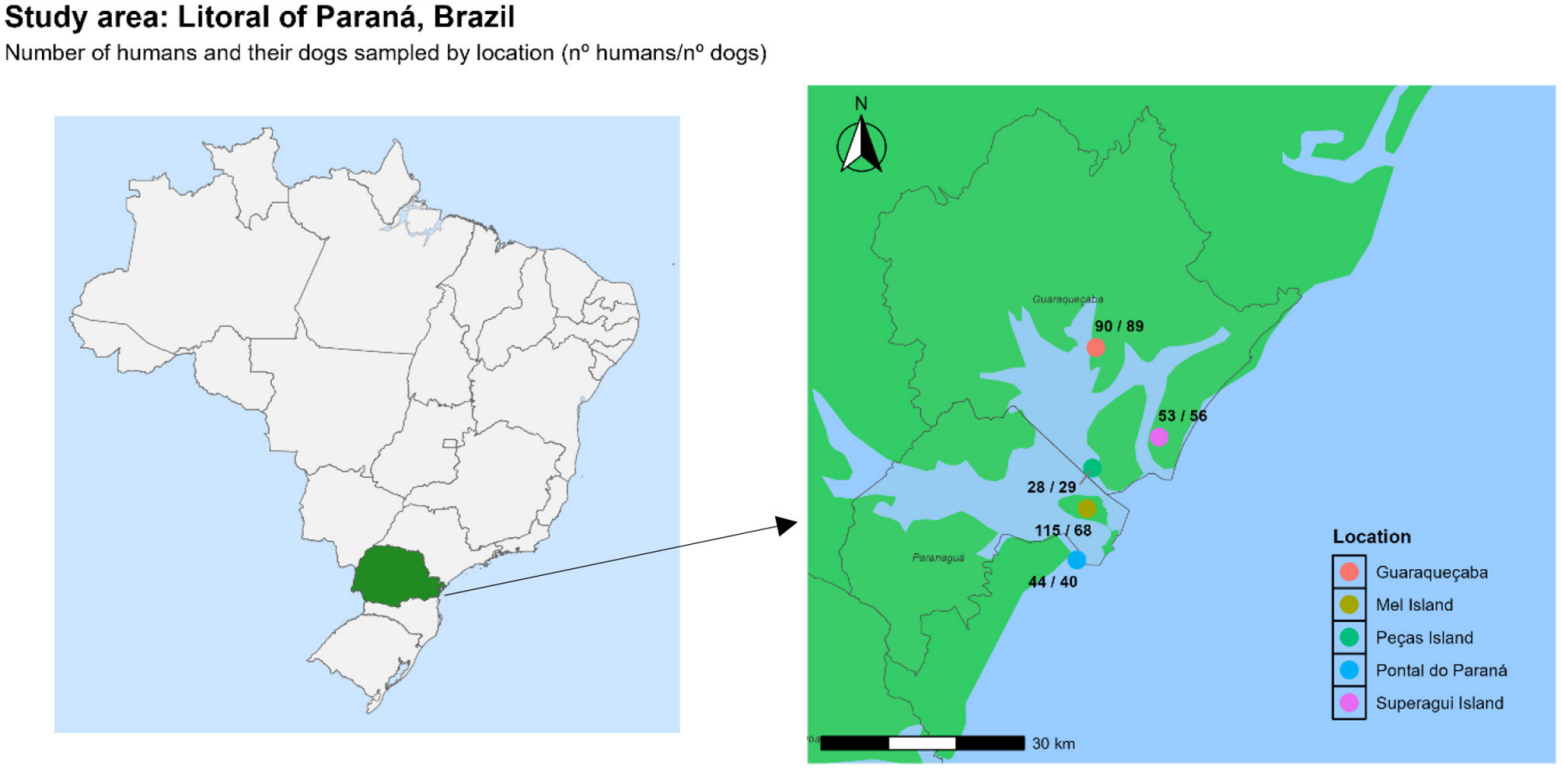
Study area of Paraná state, southern Brazil presenting the sampling locations and number of human and dog samplings in each location (R-Studio v4.4.2) (19, 20).

In addition to these three islands, samples were also collected in the mainland seashore cities of Pontal do Paraná (25°4′01.2″S and 48°1′26.6″W) with 30,425 habitants and Guaraqueçaba (25°7′47.9″S and 48°9′20.8″W) with 7,474 habitants, both located nearby the islands and used as mainland ports for sea transportation and commerce with the three islands. These two cities were also used herein for prevalence comparison between mainland and oceanic islands (Figure 1) (15).

### 2.4 Sample collection

In the present study, human and dog blood samplings were performed by convenience. Human blood samples were collected by cephalic vein puncture after signing a consent form and completing an epidemiological questionnaire, with the procedures being performed by certified physicians and nurses at the local Primary Care Units. Dog blood samples were obtained by jugular puncture performed by qualified veterinarians, after the owner signed a consent form and completed an epidemiological questionnaire. All blood samples were placed in sterile tubes containing a serum separator gel without an anticoagulant, centrifuged at 1,500 RPM for 5 min, and the serum stored at −20°C until processing.

A minimum sample size of 292 participants was estimated to assess the proportion of Coxiella seropositivity in humans, assuming an absolute precision of ± 2.5%. Considering a potential 10% loss due to non-responses or losses, the final sample size was set at 325. The calculation was based on the Wald method, with a 95% confidence level and an expected seropositivity rate of ~5% (16). The estimation was performed using the epiR package in RStudio v.2025.05.0 (17).

### 2.5 Serological testing

Human serum samples were analyzed using an in-house indirect immunofluorescence assay (IFA) developed and validated in Brazil, as previously described by Horta et al. (18). The assay employed *C. burnetii* strain At12, originally isolated from *Amblyomma tigrinum* ticks in Argentina, and maintained through successive passages in Vero cell monolayers under biosafety level 3 (BSL-3). Infected Vero cells, containing a mixed antigenic profile of phase I and phase II forms of *C. burnetii*, were fixed onto glass slides as the antigen substrate. Serum samples were screened at an initial dilution of 1:64 using fluorescein isothiocyanate (FITC)-conjugated anti-human IgG antibodies. Previously characterized positive and negative sera were included as internal controls in each assay run. Samples exhibiting specific fluorescence were titrated by serial two-fold dilutions, with the endpoint titer defined as the highest dilution showing clear fluorescence (18).

Dog serum samples were tested by the same in-house indirect immunofluorescence assay (IFA) protocol used on human samples, except for the use of anti-dog IgG fluorescein isothiocyanate (FITC) antibody (Zoonosis Control Center, São Paulo, Brazil). Dog samples previously tested during the laboratory routine were used as positive and negative controls. The positive samples were further tested to serial dilutions and titrated according to the last dilution in which luminescence was observed.

## 3 Results

In overall, 5/335 (1.49%) humans were seropositive to anti-*C. burnetii* antibodies, with 4/197 (2.0%) seropositive individuals from the islands and 1/138 (0.7%) from the mainland seashore areas (Figure 1; Tables 1,2).

**Table 1:** Human and canine serum samples tested for *Coxiella burnetii* in island and seashore mainland areas of southern Brazil, with coordinates and seropositivity (%).

**Locality**	**Geographical coordinate**	**Human samples (positivity %)**	**Dog samples (positivity %)**
Mel island	25°2′32.4″S, 48°8′15.1″W	115 (2/1.7%)	123 (0/0%)
Peças island	25°7′36.5″S, 48°0′06.5″W	27 (1/3.7%)	29 (0/0%)
Superagui island	25°7′27.1″S, 48°4′43.6″W	55 (1/1.8%)	60 (0/0%)
Guaraqueçaba	25°7′47.9″S, 48°9′20.8″W	93 (0/0%)	91 (0/0%)
Pontal do Paraná	25°4′01.2″S, 48°1′26.6″W	45 (1/2.2%)	58 (0/0%)

**Table 2:** Epidemiological data and serological results for *C. burnetii* in island and seashore mainland areas of southern Brazil.

	**IFA result** ***Coxiella burnetti***	
**Variable**	**Positive (%)**	**Negative (%)**
**Local**		
Seashore	1 (0.7)	133 (99.3)
Islands	4 (2%)	192 (98%)
**Sex**		
Female	4 (1.9)	203 (98.1)
Male	1 (0.8)	122 (99.2)
**Age**		
0–19	0 (0)	30 (100)
20–39	2 (1.4)	141 (98.6)
40–59	3 (2.5)	117 (97.5)
60+	0 (0)	35 (100)
**Dogs in the house**		
No	1 (2.9)	33 (97.1)
Yes	4 (1.4)	292 (98.6)
**Cats in the house**		
No	3 (1.5)	203 (98.5)
Yes	2 (1.6)	121 (98.4)
**Raw meat consumption**		
No	2 (0.9)	221 (99.1)
Yes	3 (2.8)	103 (97.2)
**Ingestion of drinkable water**		
No	2 (1.8)	112 (98.2)
Yes	3 (1.4)	213 (98.6)
**Presence of rodents**		
No	2 (1.4)	144 (98.6)
Yes	3 (1.6)	180 (98.4)
**Presence of flood**		
No	1 (0.7)	134 (99.3)
Yes	4 (2.1)	191 (97.9)

Out of the 212/352 (60.2%) dogs sampled on islands and 149/352 (42.3%) dogs sampled on seashore mainland, no dog sample was found seropositive in the present survey (Tables 1,2).

## 4 Discussion

To the authors knowledge, this was the first concomitant Q fever serosurvey in owners and their dogs living in oceanic islands and mainland seashore areas. This study has shown relatively lower owner and dog seropositivity for *C. burnetii*, which may be explained by geographical isolation and distance from livestock due to environmentally protected areas, even under potential exposure to local wildlife fauna.

Although the survey herein may have been impacted by geographical isolation, several other oceanic islands worldwide have reported *C. burnetti* infection and exposure, including 590 notified cases in Canary Islands, Spain between 2016 and 2022 (3.93 per 100,000 habitants-year) (21), 21/241 (8.7%) seropositive inhabitants of Reunion Island, France (22), 152/3,300 (4.6%) reagent individuals from Crete, Greece (23), and 41/98 (41.8%) seropositive veterinary students from Caribbean island of St. Kitts, suggesting occupational risk (24). In addition, 8/46 (17.4%) dogs were positive by molecular analyses in Guadeloupe, a French territory (25). Although wildlife interaction has been previously indicated to increase *C. burnetii* seroprevalence (26–28), the low rate of human and dog seropositivity found herein may reflect a low infection rate of *C. burnetti* in wildlife.

In Brazil, human *C. burnetii* infection has been most associated with livestock and environmental contamination (5, 16, 29–31). The low seroprevalence reported on islands and seashore herein may be consequence of livestock absence (prohibited in environmentally protected areas) and geographical isolation. In addition, as the longest continuous strip of Atlantic Forest in Brazil, the seashore rainforest (Serra do Mar) mountains may have also acted as natural barriers against airborne dispersal of *C. burnetii* in such areas. Moreover, lack of dog seropositivity herein may have also suggested that seropositive humans were exposed to *C. burnetti* outside islands and seashore mainland areas. Thus, results herein have contrasted to *C. burnetti* higher exposure in other Brazilian settings including rural *quilombola* communities, female penitentiary, parks and zoos of southern Brazil, which may have presented higher contact to livestock and wildlife (8, 9).

Such low or absence of disease pattern due to geographical isolation has been proposed and called as “island effect”, in which isolated populations such as traditional fishermen communities may have no spreading of certain infectious diseases due to a lack of pathogen source, as the relatively low seroprevalence observed herein for Q fever with 1.49% for humans and 0% for dogs, and previously for toxoplasmosis with 18.0% for human and 23.3% for dogs (32), both likely due to a lack of nearby livestock farming. On the other hand, such “island effect” may exacerbate disease prevalence due to multiple infection routes and pathogen sources, associated to continuous daily exposure within a closed environment, as reported for toxocariasis with 64.6% seroprevalence in humans and 10.43% positive feces samples of dogs (33).

As limitation of the present study, associated risk factors could not be properly assessed and tested due to the low seropositivity found herein. In addition, as the present study have solely surveyed islands and seashore areas of southern Brazil, further studies should also focus on owners and dogs living on other Brazilian seashore regions and islands with livestock farming (not environmentally protected), which may provide distinct serological patterns. Finally, considering that vulnerable populations have historically faced barriers in accessing healthcare services, often having less knowledge about emerging diseases and their impact (34–36), active disease surveillance should be continuously performed in such vulnerable settings. As wildlife have been considered as a potential source of *C. burnetii* transmission in nearby areas of conservation units (3), further studies should also considerer wildlife surveying. Also, travel history of seropositive individuals may be useful to clarify areas of transmission.

Although the in-house IFA used in this study employed a mixed phase I/II antigen, which does not allow phase-specific interpretation of the immune response, it was adequate for detecting previous exposure to *C. burnetii*. The use of phase-differentiated serology could provide additional insights into the stage and origin of infection, particularly in distinguishing chronic cases potentially acquired outside the study area. However, such tests rely on commercial kits that are not available in Brazil.

As another limitation, some dogs may have been previously exposed to *C. burnetii* and later experienced a decline in antibody levels to below detectable thresholds. While this seroconversion phenomenon has been well described in humans, the kinetics of IgG decline in dogs remains to be fully understood (37). Thus, although previous exposure cannot be entirely ruled out, the absence of seropositive dogs in a substantial sampling has supported the interpretation of limited or absent *C. burnetii* circulation in the studied environments.

The present study has shown low *C. burnetti* seropositivity in human and dog populations living on islands and seashore mainland areas in southern Brazil. Such low seroprevalence may have been consequence of livestock absence on environmentally protected islands, associated with geographical isolation and natural barrier against airborne dispersal due to rainforest seashore mountains. In addition, the absence of dog seropositivity herein may suggest that seropositive humans were exposed to *C. burnetti* outside islands and seashore mainland areas, which may provide new insights of *C. burnetii* transmission, disease cycle and prevention.

## Data Availability

The original contributions presented in the study are included in the article/Supplementary material, further inquiries can be directed to the corresponding author.

## Supplementary material

Table S1 Antibody titers of seropositive individuals to Coxiella burnetti from islands and mainland seashore areas of Brazil.
