# Supplementary material for: One Health approach to Coxiella burnetii: first serosurvey of owners and dogs living on oceanic islands and mainland seashore areas of Brazil
Source: Front Public Health. 2025 Aug 13;13:1643457. doi: 10.3389/fpubh.2025.1643457 (PMC12400517; doi:10.3389/fpubh.2025.1643457)
Supplement: Table S1 — Antibody titers of seropositive individuals to Coxiella burnetti from islands and mainland seashore areas of Brazil. [file Data_Sheet_1.pdf]

Supplementary Material

Supplementary table 1. Antibody titers of seropositive individuals to *Coxiella burnetti* from islands and mainland seashore areas of Brazil.

Sample	*C. burnetii* seropositivity
**Human samples**	
**Mel Island**	
1H	Negative
02H	Negative
03H	Negative
04H	Negative
05H	Negative
06H	Negative
07H	Negative
08H	Negative
09H	Negative
10H	Negative
11H	Negative
12H	Negative
13H	Negative
14H	**Positive (1:64)**
15H	Negative
16H	Negative
17H	Negative
18H	Negative
19H	Negative
20H	Negative
21H	Negative
22H	Negative
23H	Negative
24H	Negative
25H	Negative
26H	Negative
27H	Negative
28H	Negative
29H	Negative
30H	Negative
31H	Negative
32H	Negative
33H	Negative
34H	Negative
35H	Negative

36H	Negative
37H	Negative
38H	Negative
39H	Negative
40H	Negative
41H	Negative
42H	Negative
43H	Negative
44H	Negative
45H	Negative
46H	Negative
47H	Negative
48H	Negative
49H	Negative
50H	Negative
51H	Negative
52H	Negative
53H	Negative
54H	Negative
55H	Negative
56H	Negative
57H	Negative
58H	Negative
59H	Negative
60H	Negative
61H	Negative
62H	Negative
63H	Negative
64H	Negative
65H	Negative
66H	Negative
67H	Negative
68H	Negative
69H	Negative
70H	Negative
71H	Negative
72H	Negative
73H	Negative
74H	Negative
76H	Negative
77H	Negative
79H	Negative
80H	Negative
81H	Negative
82H	Negative

83H	Negative
84H	Negative
85H	Negative
86H	Negative
87H	Negative
88H	Negative
89H	Negative
90H	Negative
91H	Negative
92H	Negative
93H	Negative
94H	Negative
95H	Negative
96H	Negative
97H-A	Negative
97H-B	Negative
98H	Negative
99H	Negative
100H	Negative
101H	Negative
102H-A	Negative
102H-B	Negative
102-C	Negative
103H-A	Negative
103HB	Negative
104H	Negative
105H	Negative
106H	Negative
107H	Negative
108H	Negative
109H	Negative
110H	Negative
111H	Negative
112H	Negative
113H	Negative
114H	Negative
115H	Negative
116H	Negative
117H	**Positive (1:64)**
118H	Negative
119H	Negative
120H	Negative
121H	Negative
122H	Negative
123H	Negative
124H	Negative

125H	Negative
126H	Negative
127H	Negative
128H	Negative
129H	Negative
130H	Negative
131H	Negative
132H	Negative
133H	Negative
134H	Negative
135H	Negative
136H	Negative
137H	Negative
138H	Negative
139H	Negative
140H	Negative
141H	Negative
142H	Negative
143H	Negative
144H	Negative
145H	Negative
146H	Negative
147H	Negative
148H	Negative
149H	Negative
150H	Negative
151H	Negative
152H	Negative
153H	Negative
154H	**Positive (1:64)**
155H	Negative
156H	Negative
157H	Negative
158H	Negative
**Guaraqueçaba**	
01H-A	Negative
01H-B	Negative
02H	Negative
03H	Negative
04H	Negative
05H	Negative
06H	Negative
07H	Negative
09H-A	Negative
09H-B	Negative

10H-A	Negative
10H-B	Negative
12H-A	Negative
12H-B	Negative
13H	Negative
15H	Negative
16H-A	Negative
16H-B	Negative
17H	Negative
18H	Negative
19H	Negative
20H	Negative
21H-A	Negative
21H-B	Negative
22H	Negative
27H-A	Negative
27H-B	Negative
29H	Negative
30H-A	Negative
30H-B	Negative
31H	Negative
32H	Negative
33H	Negative
34H	Negative
35H	Negative
36H	Negative
37H	Negative
39H	Negative
40H-A	Negative
40H-B	Negative
42H	Negative
43H	Negative
44H	Negative
46H	Negative
47H	Negative
48H	Negative
50H	Negative
52H	Negative
54H-A	Negative
54H-B	Negative
55H	Negative
56H	Negative
58H	Negative
60H	Negative
62H	Negative
63H	Negative

66H	Negative
67H	Negative
68H	Negative
69H	Negative
70H-A	Negative
70H-B	Negative
71H	Negative
72H	Negative
73H	Negative
74H	Negative
76H	Negative
77H	Negative
78H	Negative
79H-A	Negative
79H-B	Negative
80H	Negative
82H	**Positive (1:128)**
83H-A	Negative
83H-B	Negative
84H	Negative
85H	Negative
86H	Negative
87H	Negative
88H	Negative
89H	Negative
90H	Negative
91H	Negative
92H	Negative
93H	Negative
95H-A	Negative
95H-B	Negative
97H	Negative
99H	Negative
100H	Negative
101H	Negative
102H	Negative
103H	Negative
104H	Negative
105H	Negative
106H	Negative
107H	Negative
108H	Negative
109H	Negative
110H	Negative
111H	Negative

112H	Negative
113H	Negative
**Superagui Island**	
01H	Negative
02H	Negative
03H	Negative
05H	Negative
06H	Negative
07H	Negative
10H	Negative
14H	Negative
15H	Negative
16H-A	Negative
16H-B	Negative
17H	Negative
18H	Negative
19H	Negative
20H	Negative
22H	Negative
23H	Negative
24H	Negative
26H	Negative
28H	**Positive (1:64)**
29H	Negative
30H	Negative
31H	Negative
32H	Negative
35H	Negative
36H	Negative
37H	Negative
38H	Negative
39H	Negative
43H	Negative
44H	Negative
48H-A	Negative
48H-B	Negative
49H	Negative
51H	Negative
52H	Negative
53H	Negative
55H	Negative
56H	Negative
57H	Negative
61H	Negative
62H	Negative
63H	Negative

64H	Negative
66H	Negative
67H	Negative
68H	Negative
69H	Negative
72H	Negative
74H	Negative
75H	Negative
77H	Negative
78H	Negative
79H	Negative
80H	Negative
81H	Negative
82H	Negative
83H	Negative
84H	Negative
85H	Negative
86H	Negative
87H	Negative
88H	Negative
**Peças Island**	
1H	Negative
2H	Negative
3H	Negative
4H	Negative
5H	Negative
6H	Negative
7H	Negative
8H	Negative
9H	Negative
